# Jaw bone metastasis from Lung cancer as sole primary source: A systematic review

**DOI:** 10.4317/jced.59554

**Published:** 2022-07-01

**Authors:** Sonia Gupta, Manveen-Kaur Jawanda, Aruna Ganganna, Suman Basavaraju, Neha Kashav, Jayata Dhawan, Sumit-Kumar Yadav, Achla-Bharti Yadav

**Affiliations:** 1 MDS, Reader, Department of Oral Pathology and Microbiology & Forensic odontology, Rayat and Bahra Dental college and hospital, Mohali, Punjab, India; 2 MDS, Professor & Head, Department of Oral Pathology and Microbiology & Forensic odontology. Laxmi bai institute of dental sciences and hospital, Patiala, Punjab, India; 3 MDS, Assistant Professor, Department of Periodontology, JSS Dental College and Hospital, Mysore, Karnataka, India; 4 MDS, Reader, Department of Periodontology, JSS Dental College and Hospital, Mysore, Karnataka, India; 5 MDS, Senior Lecturer, Department of Pediatric and Preventive Dentistry, Bhojia Dental College and Hospital, Baddi, HP, India; 6 MDS, Senior Lecturer, Department of Pediatric and Preventive Dentistry, MM College of Dental Sciences and Research, Mullana, Haryana, India; 7 MDS, Professor, Department of Orthodontics & Dentofacial Orthopedics, D J College of Dental Sciences & Research, Modinagar, UP, India; 8 MDS, Professor, Department of Oral Pathology & Microbiology, D J College of Dental Sciences and Research, Modinagar, UP, India

## Abstract

**Background:**

Lung cancer is one of the leading causes of death worldwide. Lung cancer metastasis to oral region is very rare. Very few research work has been conducted till date to analyse the jaw bone metastasis from Lung cancer as the primary source. The goal of this research was to examine published cases of jaw bone metastasis from lung cancer as the sole primary source from 1st December 1961 to 31st December 2021 and to learn about their characteristics.

**Material and Methods:**

An electronic search of the published English literature was performed in PubMed/ Medline, Scopus, Google Scholar, and Research gate databases, using keywords like ‘Lung cancer’, OR/AND ‘Lung carcinoma’, OR/AND ‘Metastasis’, OR/AND ‘Primary’, OR/AND ‘Source’, OR/AND ‘Oral cavity’ OR/AND ‘Jaw’, OR/ AND ‘Mandible’, OR/AND ‘Maxilla’, OR/ AND ‘Temporomandibular joint’, OR/ AND ‘Condyle’, OR/ AND ‘Ramus’, OR/ AND ‘Maxillary sinus’, AND Initial’, OR/ AND ‘Treatment’, OR/AND ‘Prognosis’, OR/ AND ‘Follow-up’, OR/AND ‘Recurrence’, OR/ AND ‘Survival rate’. We also searched all related journals manually. Reference list of all articles was also checked. Data extracted were tabulated and summarized.

**Results:**

In total, we found 60 relevant publications with 66 patients in our research. The prognosis was poor, with a survival time of 1 week to 1.5 years. The most prevalent diagnosed metastatic lung cancer to jaw bones was adenocarcinoma and mandible was the predominant site.

**Conclusions:**

Jaw bone metastasis from lung cancer is rare and has a bad prognosis. Because of their resemblance to other jaw problems and late clinical signs, these lesions go unnoticed the majority of the time, making detection difficult. More cases need to be published in order to raise awareness of these lesions and gain a better understanding of their characteristics.

** Key words:**Jaw bone, lung cancer, metastasis, primary, prognosis.

## Introduction

According to GLOBOCAN databases, Lung cancer (LC) has overtaken breast cancer as the 2nd most often diagnosed cancer worldwide, and it remains one of the major causes of mortality (1). In 2020, an estimated 2.2 million new cases of LC were diagnosed worldwide, contributing for around 11.4 % of the global cancer burden (1). LC is characterised by its stealthy nature, remaining asymptomatic until the disease has progressed to an advanced stage, which is associated with a risk of distant metastasis. And, most of the time, even once symptoms arise, patients disregard them, resulting in a delay in diagnosis and treatment (2). The liver, kidney, adrenals, brain, skeletal muscles, vertebrae, and other organs are all involved in distant distribution via LC (3). According to a retrospective analysis done by Tsuya *et al*. in 2007, bone metastasis from LC is a frequent event and the most common bone metastasized from LC is spine followed by the ribs, ilium, sacrum, femur, humerus, scapula and sternum (4). LC metastasis into the oral cavity is uncommon, and mostly affects oral soft tissues rather than jaw bones (JB). Few cases of jaw bone metastasis (JBM) from LC as the sole primary source have been reported in the literature. And the prognosis for such cases is poor, indicating the critical importance of their early identification and management. Due to their strong resemblance to benign growth, late appearance, or lack of interpretation, diagnosis of JBM remains difficult for clinicians and pathologists (5). The goal of this research was to examine published cases of JBM from LC as the sole primary source from 1st December 1961 to 31st December 2021, , as well as to learn about their characteristics.

## Material and Methods

The Preferred Reporting Items for Systematic Reviews and Meta-Analyses (PRISMA) standards were used to conduct this research. There was no need to seek any ethical approval because of the nature of the current review.

-Focused PECO question

For search screening, we framed a focused PECO question; How many cases of JBM from LC as the sole primary source have been documented in the literature and what is the prognosis of these metastatic lesions?

Population: Patients with JBM from LC

Exposure: LC metastasis

Comparison: Not applicable for this research

Outcome: Prognosis of JBM from LC

**Figure 1:**
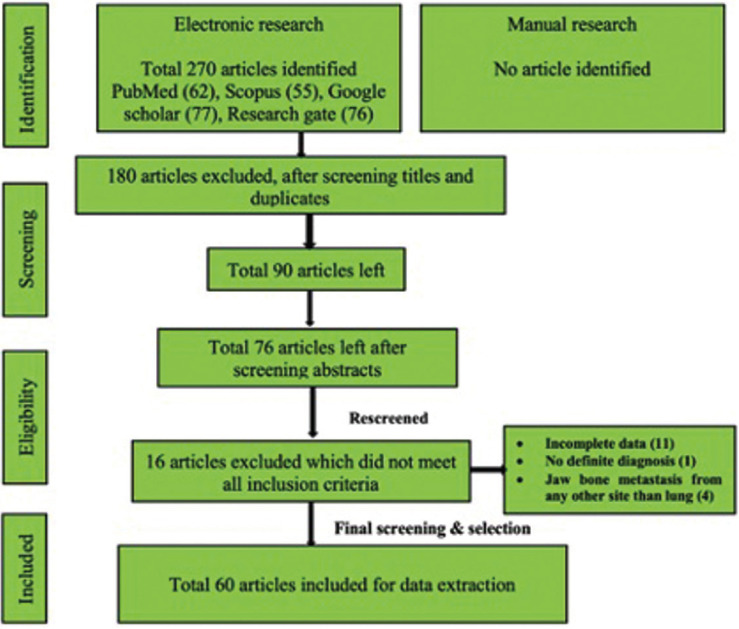
PRISMA Flow chart showing search strategy and screening.

-Search strategy for identification of studies (Fig. 1)

An electronic search of the published English literature was performed in PubMed/ Medline, Scopus, Google Scholar, and Research gate databases, using keywords like ‘Lung cancer’, OR/ AND ‘Lung carcinoma’, OR/AND ‘Metastasis’, OR/AND ‘Primary’, OR/AND ‘Source’, OR/AND ‘Oral cavity’ OR/AND‘Jaw’, OR/ AND ‘Mandible’, OR/AND ‘Maxilla’, OR/ AND ‘Temporomandibular joint’, OR/ AND ‘Condyle’, OR/ AND ‘Ramus’, OR/ AND ‘Maxillary sinus’, OR/ AND ‘Initial’, OR/ AND ‘Treatment’, OR/AND ‘Prognosis’, OR/ AND ‘Follow-up’, OR/AND ‘Recurrence’, OR/ AND ‘Survival rate’ We also searched all related journals manually. Reference list of all articles was also checked.

-Screening of studies

The current review involved three steps screening of the studies. In the first step, titles were reviewed by two authors (SG, MKJ) independently and duplicates were removed. Then two authors ( AG, SB) reviewed the selected abstracts of all the reports independently. In the final stage, the text of selected studies was screened by remaining four authors separately (NK, JD, SKY, ABY). Full report was collected, discussed, and resolved among all authors for cases that appeared to fit the inclusion criteria or for which evidence was insufficient to make a clear determination.

-Inclusion criteria

• Confirmed cases of JBM from LC as the sole primary source. Papers included were from 1st December 1961 to 31st December 2021.

• Type of studies: Case reports, letter to editor, Retrospective analysis and correspondence.

• In retrospective analysis, only those cases were selected in which LC was the sole primary source of JBM.

• Cases were selected beyond the restriction of limitations on parameters such as age, gender, ethnicity or socioeconomic status, etc.

• Articles published only in English language were included.

-Exclusion criteria

• Cases with no definite diagnosis of JBM from LC as the sole primary source.

• Publications reporting the JBM from any other site than lung.

• Cases of oral soft tissue metastasis from LC as the primary source.

• Epidemiological studies, case control studies, cohort studies which lack individual patient data, were excluded.

• Review articles, editorials, conference abstracts, hypothesis papers, web news, media reports, animal studies.

• Duplicate, irrelevant and incomplete data were excluded.

• Articles published in languages other than English were excluded.

-Outcome measures

1. Primary outcome measures: To evaluate the number of cases of JBM from LC as the sole primary source reported in the literature from 1st December 1961 to 31st December 2021, and to determine their prognosis.

2. Secondary outcome measures: To evaluate factors such as.

◦World-wide distribution of cases of JBM from LC 

◦Patient’s demographic details

◦Associated risk factors

◦Predominant site of JBM from LC

◦Clinical and radiographic features of these metastatic lesions

◦Most prevalent type of metastatic LC 

◦Type of therapies used to treat such metastatic lesions

-Risk of bias assessment 

Most of the studies included in this review were case reports. Risk of bias in the included studies were appraised following CARE checklist guidelines. In many of the studies, there was missing information regarding many parameters used for data extraction in our research. We tried reaching the authors of those cases to clarify this bias; however we were unable to recover the missing information.

-Data extraction & analysis

After study selection, screening and a thorough examination, the data were extracted. The information gathered was cross-checked and tabulated into three Tables ( Table 1- Table 3 cont.). In case of missing data, 6 weeks’ time was given to gather the information. If the information was still missing, we then indicated the missing data as “Not available (NA)” in the text and in the Tables. Extracted data points in Table 1 included demographic details such as; authors’ names, year of publication, country, age of patient, gender of patient, previous history of LC and associated risk factors. Table 2 included clinical details such as; jaw involved, right/left side, anterior/posterior side, chief complaint, clinical features, radiographic features, provisional diagnosis, final diagnosis, side of LC, JB as the initial site of metastasis and any other site of metastasis.  Table 3 described therapeutic parameters such as; type of treatment given, prognosis, and cause of death.

**Table 1:**
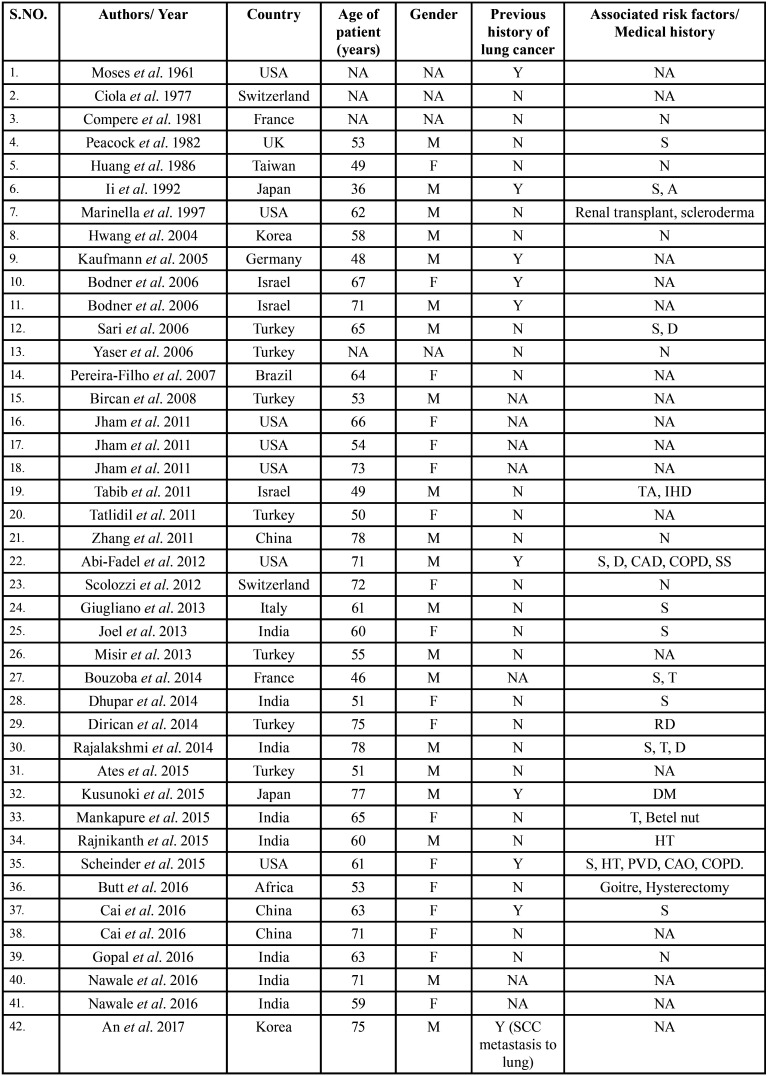
Demographic data of patients with jaw bone metastasis from lung cancer as the sole primary source (1st December 1961 to 31st December 2021).

**Table 1 cont.**
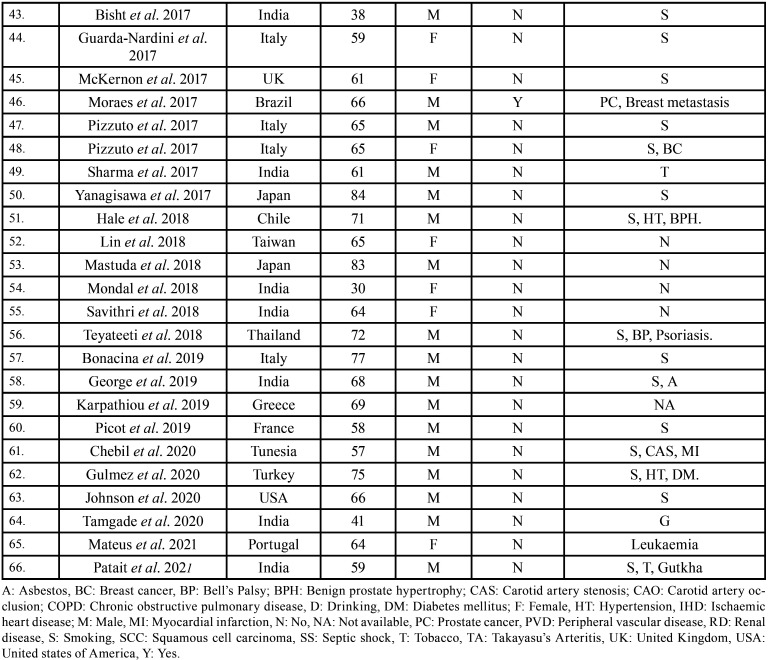
Demographic data of patients with jaw bone metastasis from lung cancer as the sole primary source (1st December 1961 to 31st December 2021).

**Table 2:**
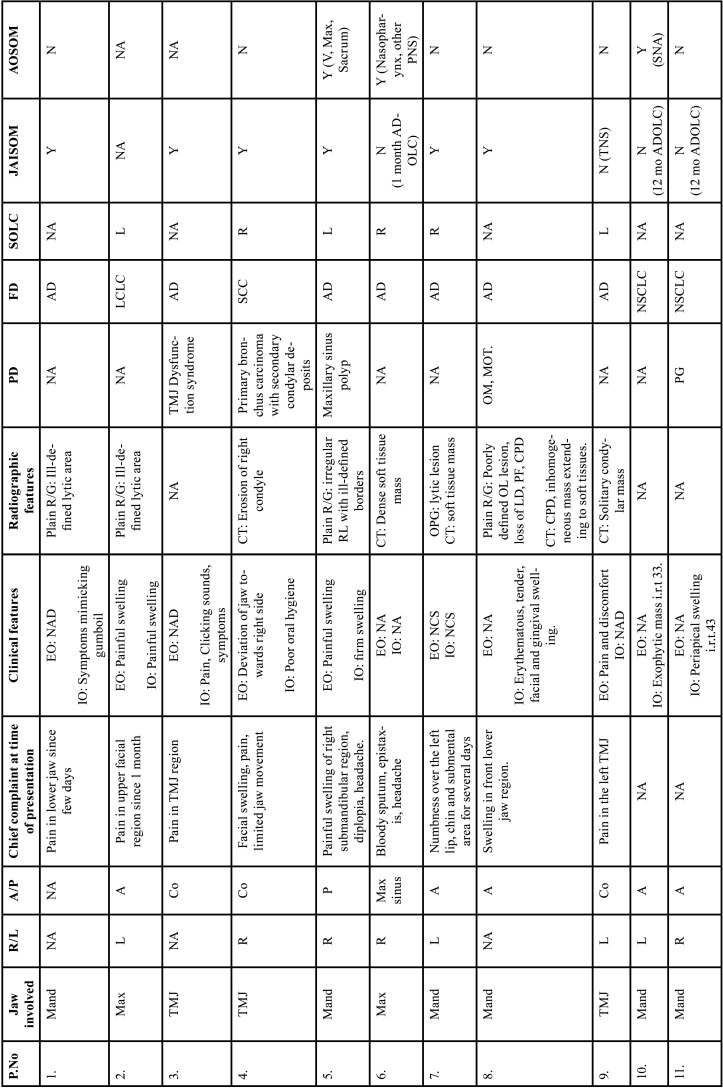
Clinical details of patients with jaw bone metastasis from lung cancer (1st December 1961 to 31st December 2021).

**Table 2 cont.**
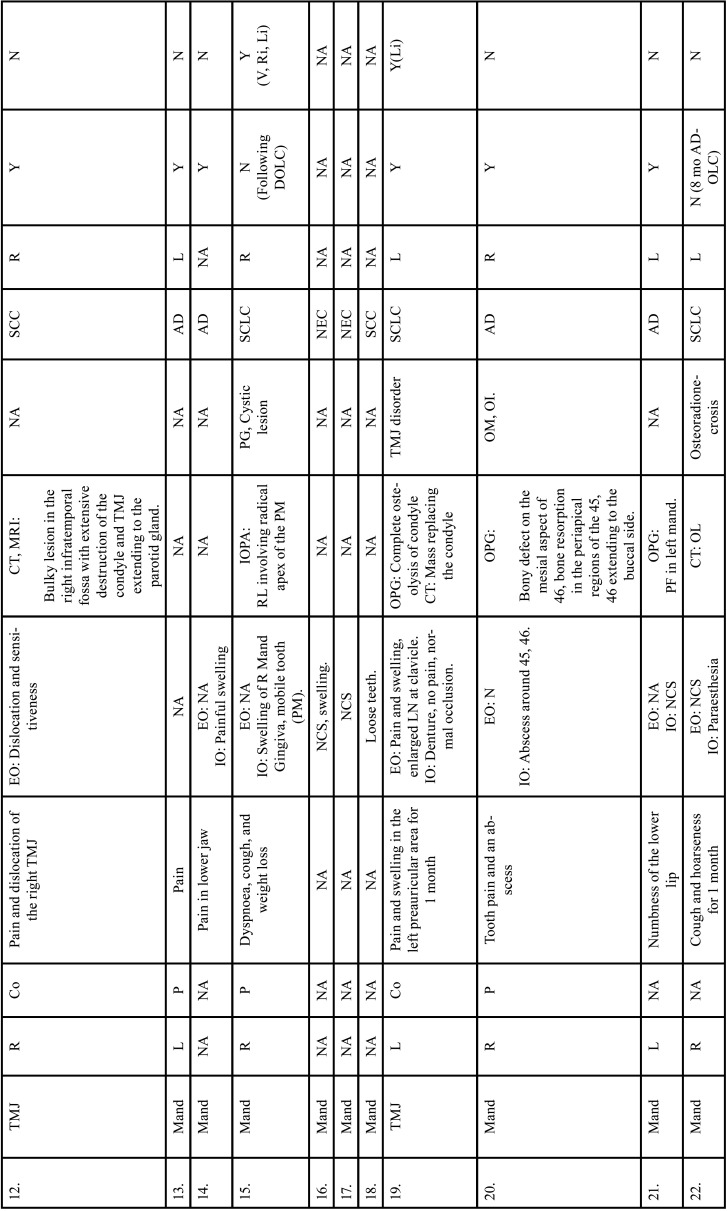
Clinical details of patients with jaw bone metastasis from lung cancer (1st December 1961 to 31st December 2021).

**Table 2 cont.-1:**
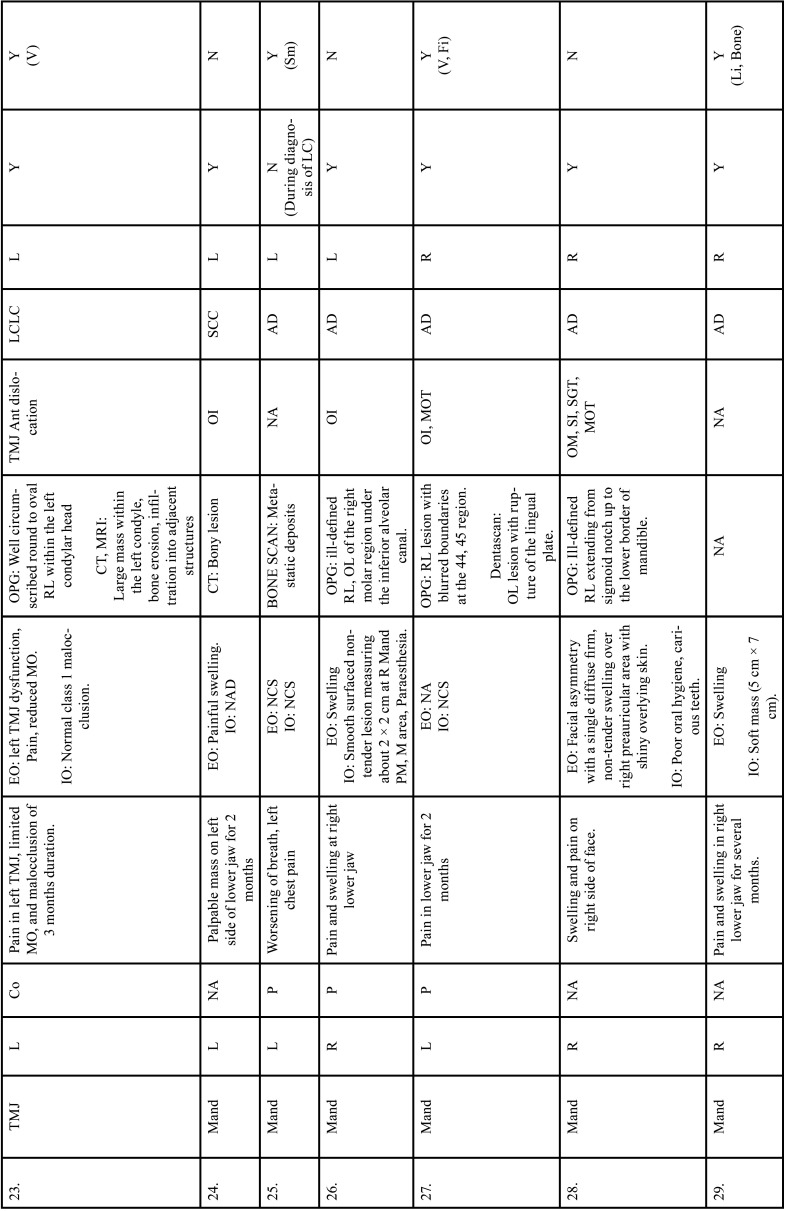
Clinical details of patients with jaw bone metastasis from lung cancer (1st December 1961 to 31st December 2021).

**Table 2 cont.-2:**
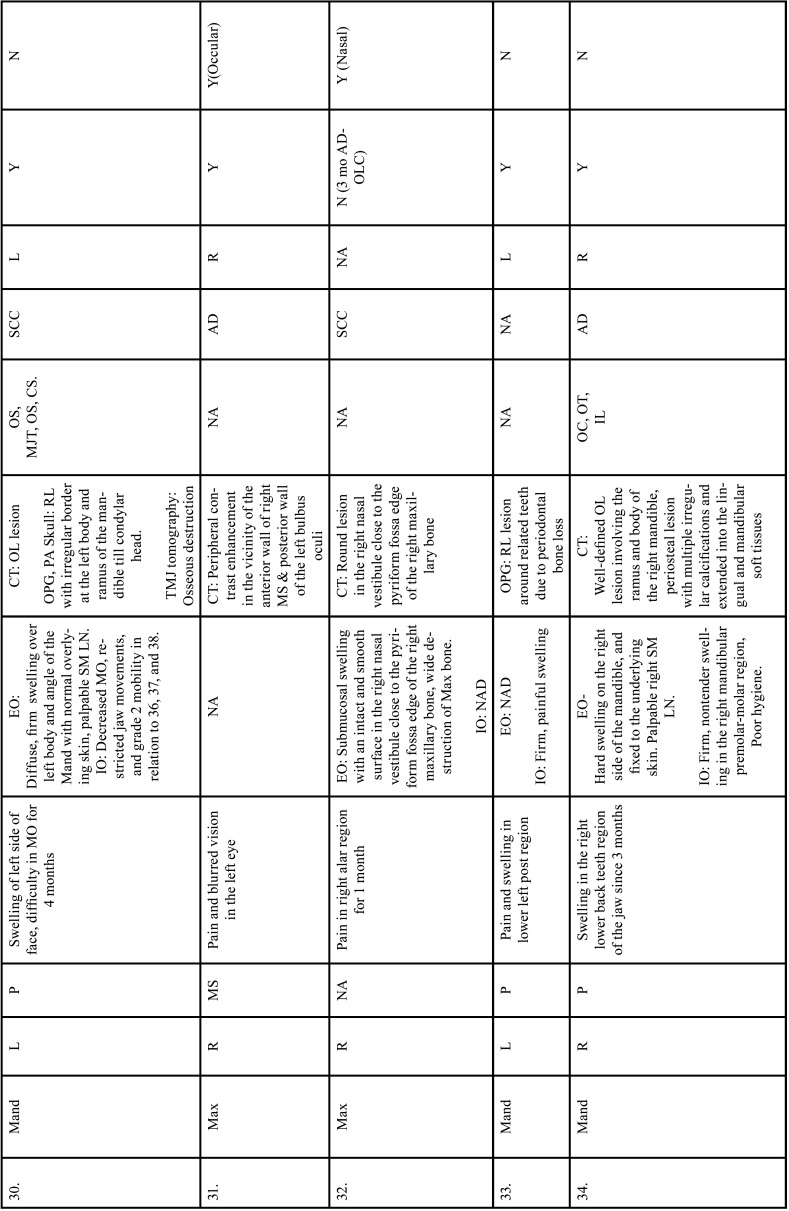
Clinical details of patients with jaw bone metastasis from lung cancer (1st December 1961 to 31st December 2021).

**Table 2 cont.-3:**
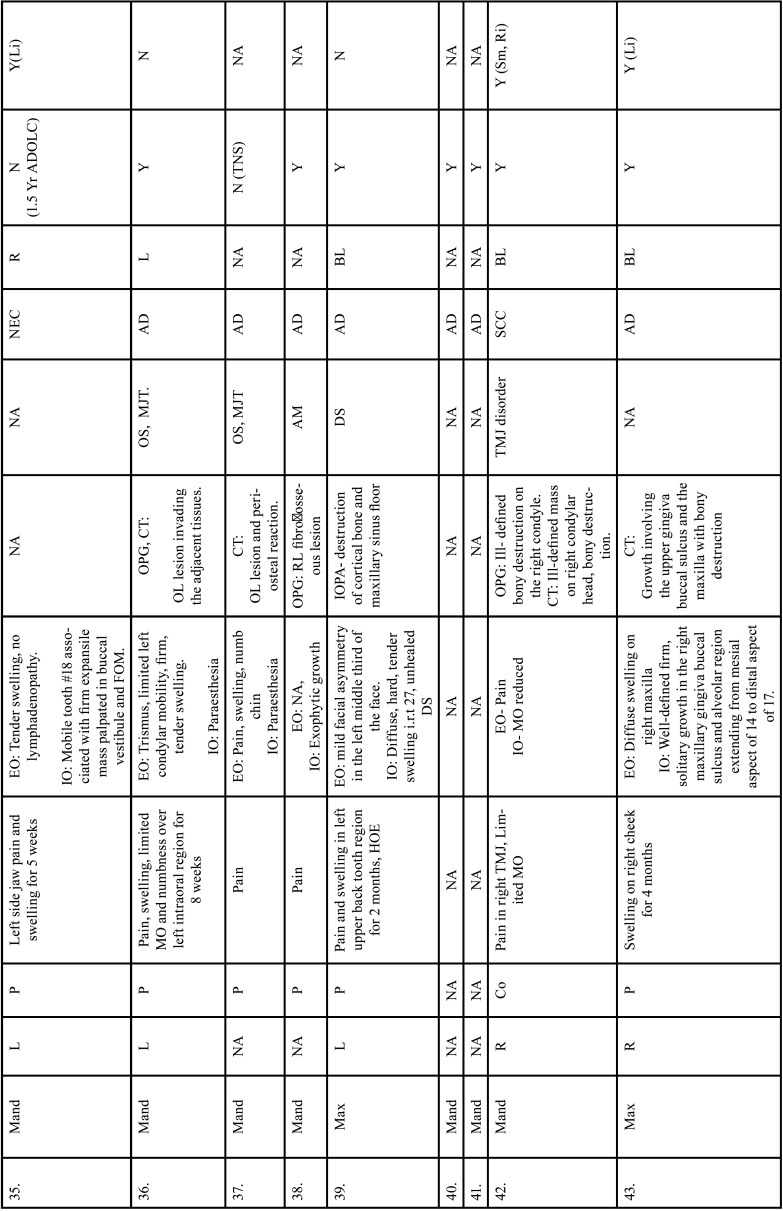
Clinical details of patients with jaw bone metastasis from lung cancer (1st December 1961 to 31st December 2021).

**Table 2 cont.-4:**
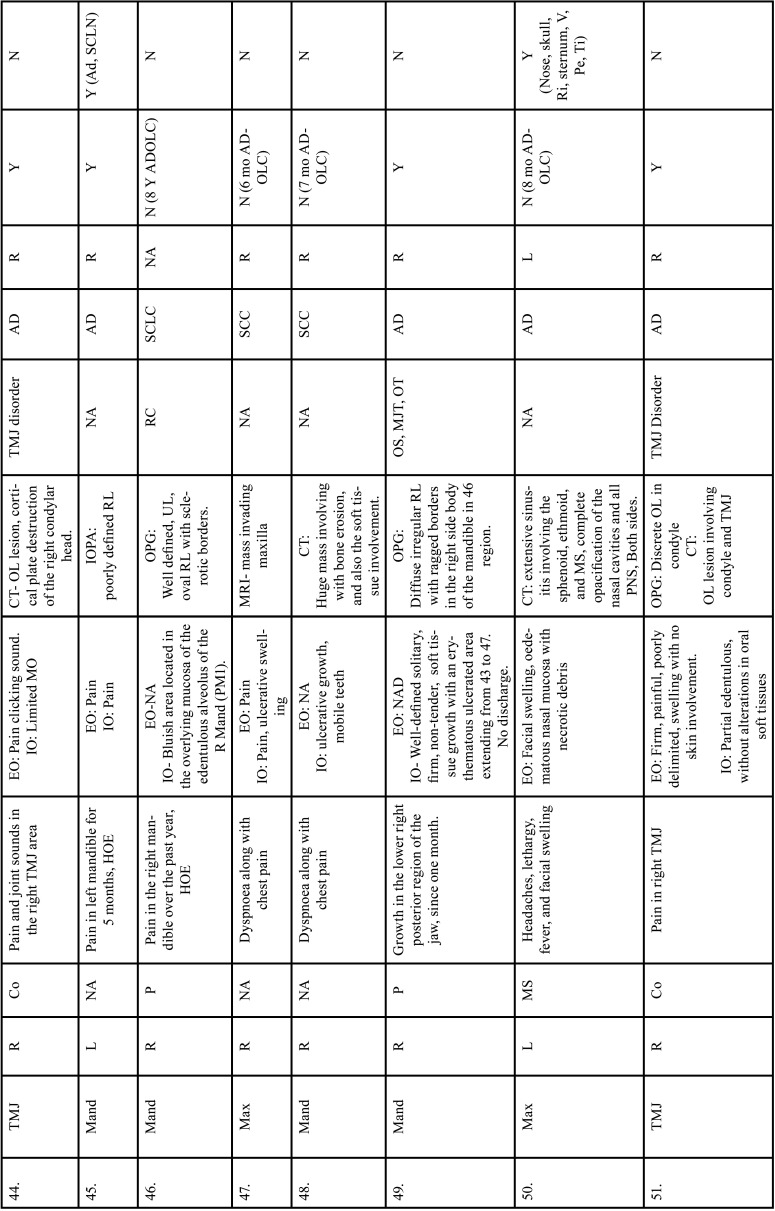
Clinical details of patients with jaw bone metastasis from lung cancer (1st December 1961 to 31st December 2021).

**Table 2 cont.-5:**
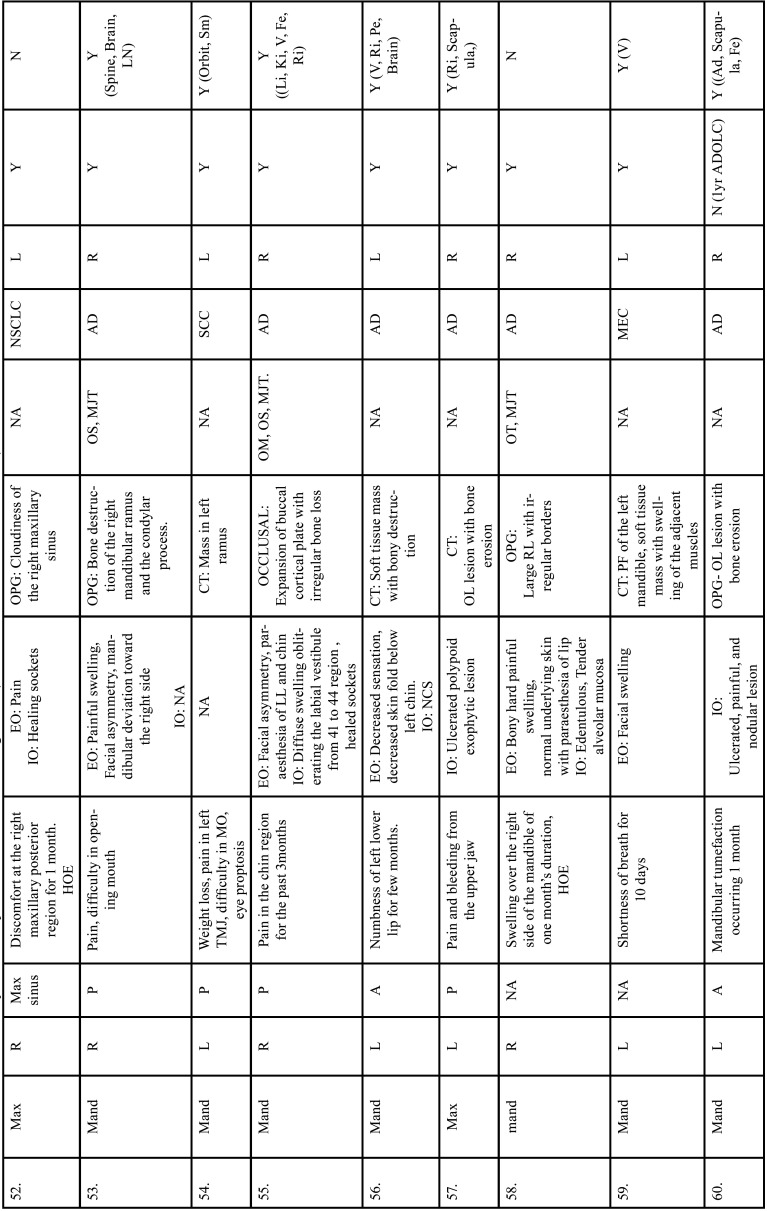
Clinical details of patients with jaw bone metastasis from lung cancer (1st December 1961 to 31st December 2021).

**Table 2 cont.-6:**
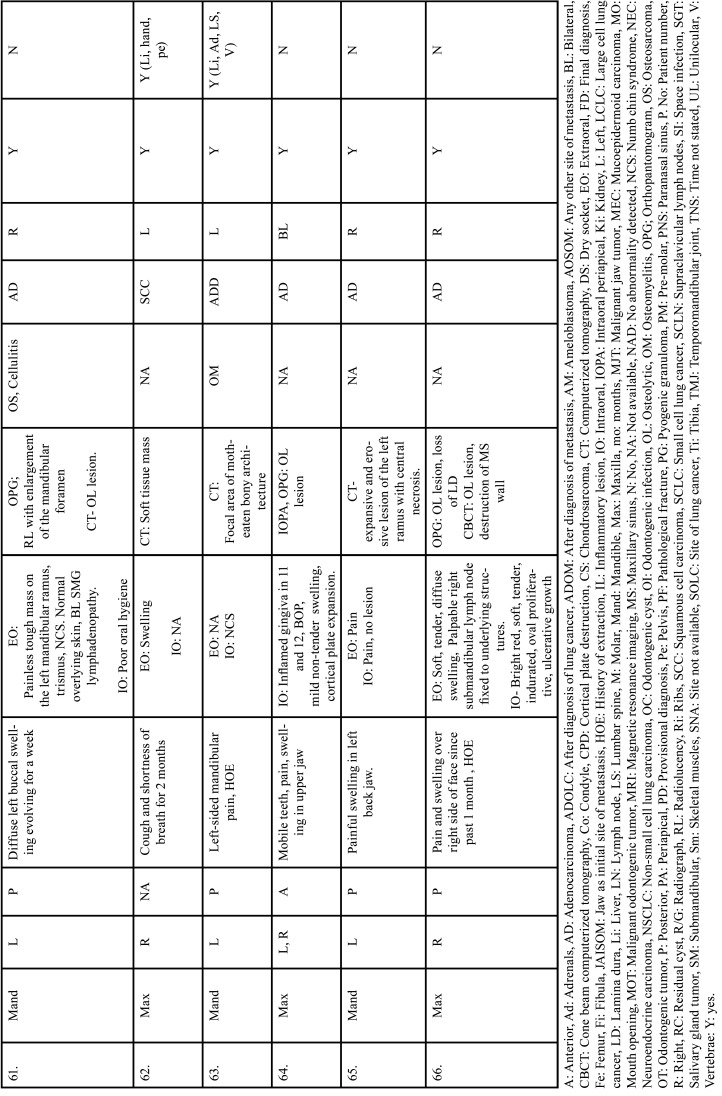
Clinical details of patients with jaw bone metastasis from lung cancer (1st December 1961 to 31st December 2021).

**Table 3:**
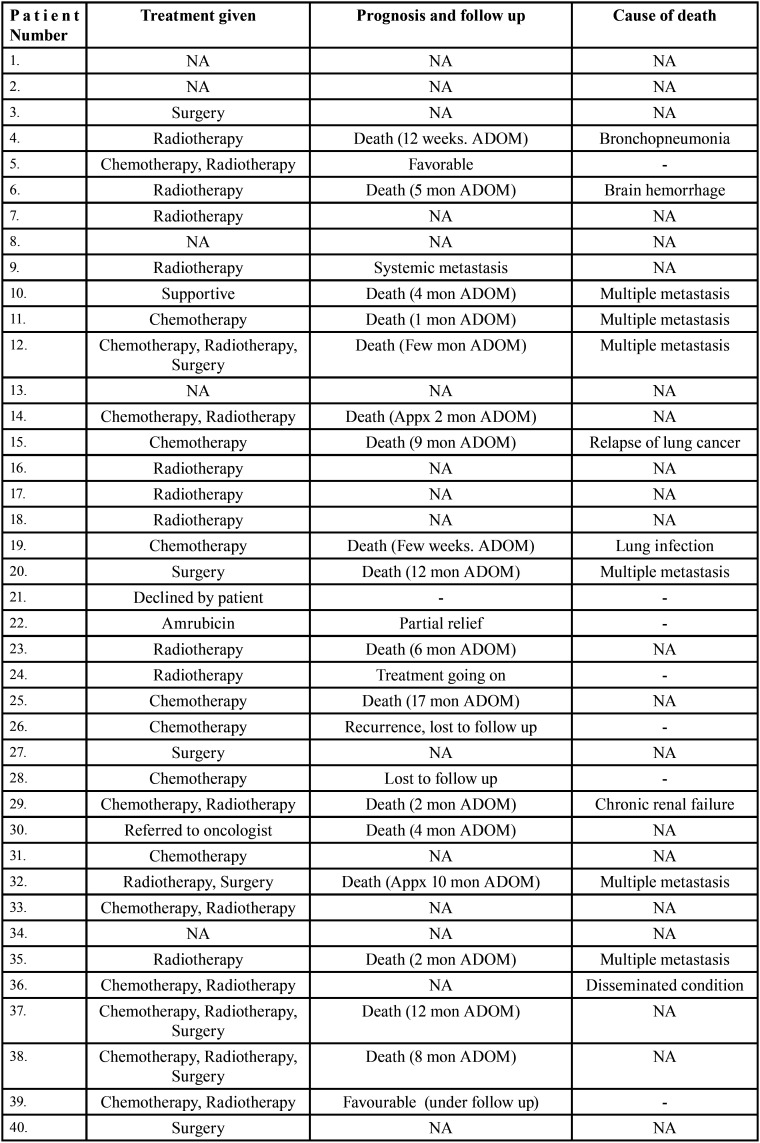
Data describing treatment and prognosis of patients with jaw bone metastasis from lung cancer (1st December 1961 to 31st December 2021).

**Table 3 cont.**
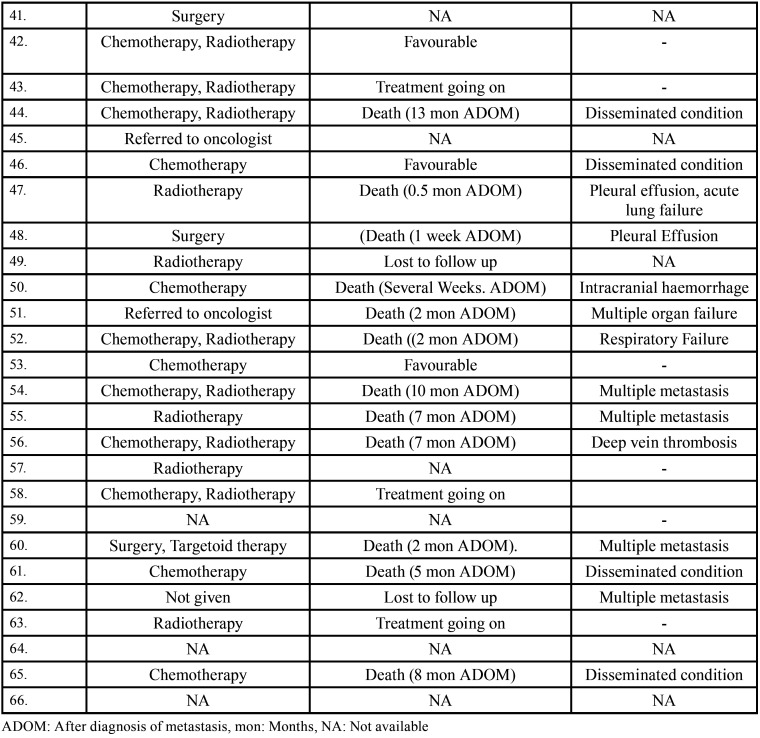
Data describing treatment and prognosis of patients with jaw bone metastasis from lung cancer (1st December 1961 to 31st December 2021).

Results ( Table 4- Table 4 cont.-1)

**Table 4:**
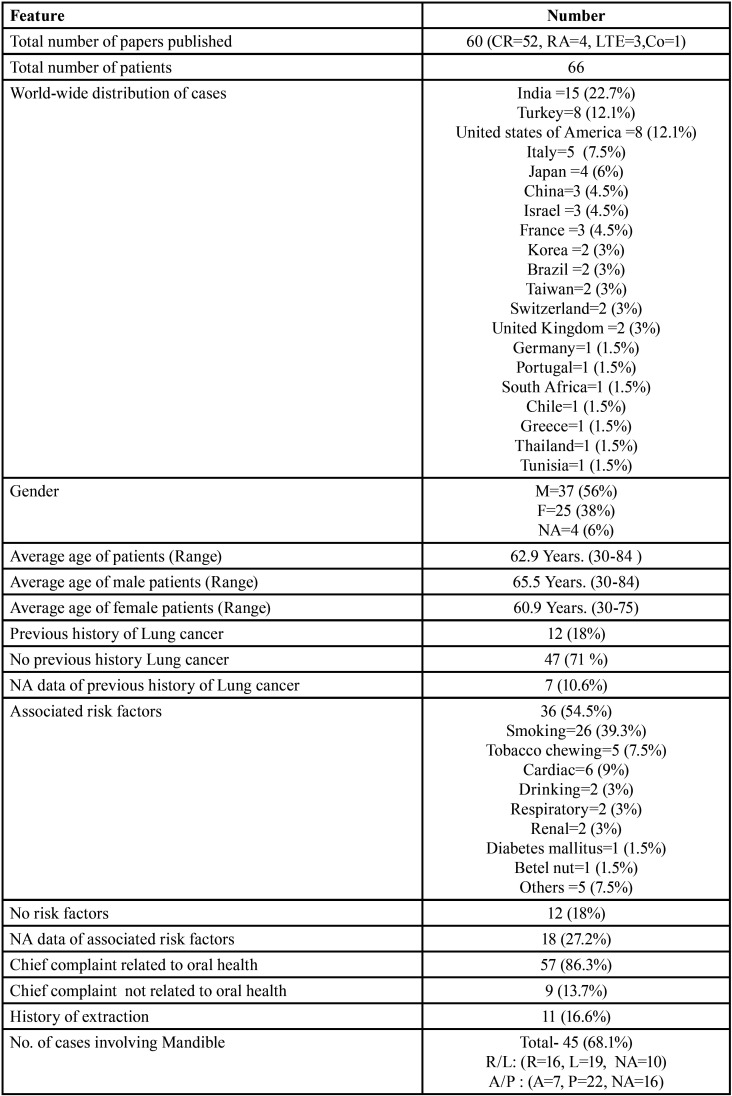
Summary of results documented from literature research describing the characteristics of jaw bone metastasis from lung cancer (1st December 1961 to 31st December 2021).

**Table 4 cont.**
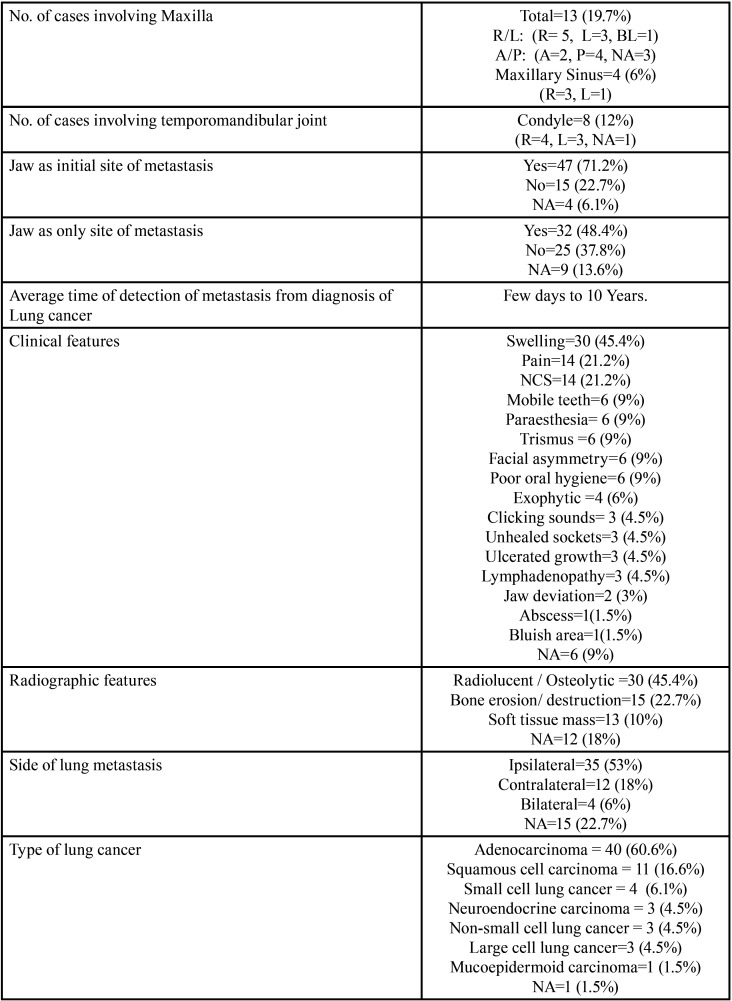
Summary of results documented from literature research describing the characteristics of jaw bone metastasis from lung cancer (1st December 1961 to 31st December 2021).

**Table 4 cont.-1:**
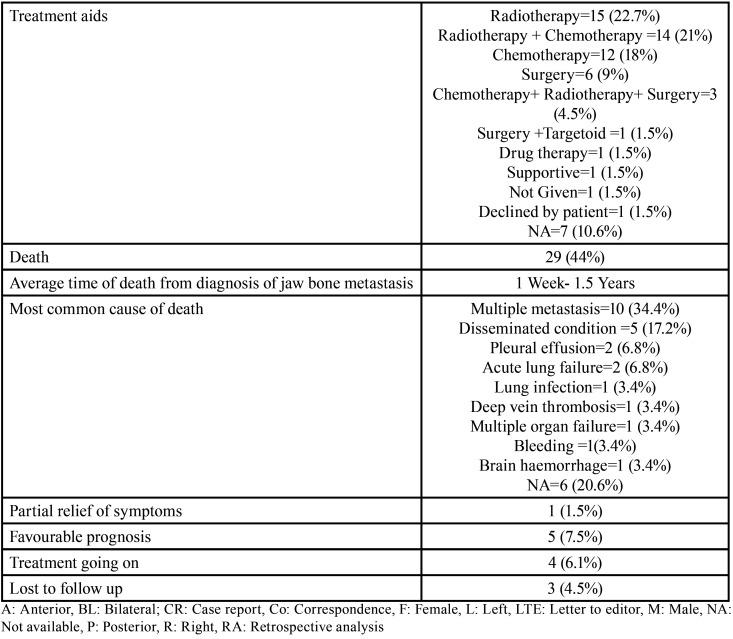
Summary of results documented from literature research describing the characteristics of jaw bone metastasis from lung cancer (1st December 1961 to 31st December 2021).

Results were expressed in descriptive statistics. Our electronic search yielded a total of 270 articles. No additional articles could be found with a manual search. After removing duplicates, screening titles and abstracts and the papers that did not fulfil the inclusion criteria, a total of 60 articles were left and included for data extraction (Fig. 1), (6-46). There were 52 Case reports, 4 Retrospective analysis, 3 letter to editor and 1 Correspondence. Some of the variable assessments in several papers, particularly in Retrospective analysis, were incomplete. There were 66 patients in total, with 37 males (56%) and 25 females (38%). The maximum number of cases were from India (n-15), followed by Turkey (n-8), USA (n-8), Italy (n-5), and Japan (n-4). The patients’ average age was 62.9 years (range 30-84 years). Mean age was 65.5 years in males and 60.9 years in females, with a range of 38-84 years and 30-75 years for males and females, respectively. 12 of the 66 patients (18%) had a previous history of LC, while the other 47 (71.2%) had none. 26 patients had a habit of smoking (39.3%), 5 had tobacco chewing habit (7.5%), 2 had drinking (3%) and 1 (1.5%) was having betel nut chewing habit. Many other underlying comorbidities were also associated. JBM was observed maximum in mandible (n-45), followed by maxilla (n-13) and Temporomandibular joint (TMJ) (n-8). Left side of mandible predominated as compared to right side and the right side of the maxilla was more involved than the left side (5 and 3 cases respectively). One case occurred bilaterally. Metastasis was seen more in the posterior region of the maxilla as well mandible. In 3 cases, this site involvement was not clear. Out of 13 maxillary cases, 4 occurred in the maxillary sinus (MS), with right sided predilection (n-3). Condylar region of TMJ was affected in 8 cases, with 4 cases on right side and 3 on left side. In 1 case, no site was given. Out of 66 cases, 57 patients (86.3%) reported with a chief complaint related to oro-dental health, while 9 (13.7%) had reported with other chief complaints. 11 patients (16.6%) had a previous history of extraction. Patients presented with variable radiographic and clinical features ( Table 4,  Table 4 cont.-1). JB was the initial site of metastasis in 47 individuals. (71.2%), while in 15 patients (22.7%), it was detected after diagnosis of LC. JB was the only site of metastasis from LC in 32 cases (48.4%), whereas 25 cases (37.8%) exhibited other parts of the body also. 35 cases (53%) showed ipsilateral metastasis, while 12 had contralateral (18%), and 4 had bilateral (6%). The average time of development of JBM from diagnosis of LC was few days to 10 years. The most common type of LC diagnosed was Adenocarcinoma (n- 40), followed by Squamous cell carcinoma (n-11). The most common treatment aids included radiotherapy (n-15), chemotherapy (n-12), and Surgery (n-6). In several cases, combined therapy was used. Even after treatment, 29 individuals (44%) died. The period between JBM diagnosis and death ranged from 1 week to 1.5 years. Results are summarized in Table  Table 4,  Table 4 cont.-1.

## Discussion

Metastasis to the oral cavity is a rare occurrence, with the real incidence unclear (1-2% of all oral cancers) (47). Because of their rarity, they are sometimes overlooked for a long time before being discovered and are diagnosed during investigations (48). According to epidemiological investigations, LC is the most common primary source of oral soft tissue metastasis, while Breast cancer is the most common source of JBM.(5) However, a few cases of JBM from LC have been recorded in the literature. In this study, we found 66 documented cases of JBM from LC.

Studies reveal that JBM affects both genders equally. In certain studies, however, a male majority was found (49). In the current study also, there was a little male predominance, with M: F =1.4:1. JBM can strike at any age, with peak incidence in 4th-7th decades (50). The patients in this study ranged in age from 3rd-8th decade.

According to researchers, smoking and tobacco consumption habits are strongly linked to the development of LC (51). Nicotine and its derivatives, which are found in tobacco and smoke, help to promote the expression of oncogenic proteins which leads to the spread of LC (52). And because these habits are more prevalent in males, they are more likely to develop LC. People with underlying comorbidities and lung disorders such as lung disorders, such as ‘chronic obstructive pulmonary disease’ are more likely to acquire LC and have a worse prognosis as a result of distant metastasis induced by a weakened immune system (50). 3% individuals in this study had respiratory comorbidities. Other most prevalent comorbities were cardiac, renal, and endocrinal (Table  Table 4,  Table 4 cont.-1.).

LC has increased in emerging countries such as India, China, Brazil, and others in recent years due to increasing smoking, drinking, and tobacco chewing habits. While in the developed and industrialized countries, the incidence rate has fallen down due to recession of these habits (52). However, the specific regional distribution of JBM from LC has not been reported in the literature. In our study, India had the highest number of cases of JBM from LC followed by Turkey and USA. Various other regions were also involved (Table  Table 4,  Table 4 cont.-1.). Looking at this data, wide region involvement of JBM from LC can be appreciated.

Pathogenic mechanisms of JBM aren’t completely understood. Metastasis is a multistage process that involves tumour cells being detached from their originating site and being transported to a secondary site via lymphatic or hematogenous channels (53). One of the proposed pathways is the “Batson’s plexus,” a valveless prevertebral venous plexus network that involves retrograde tumour cell movement from the lungs to the face (54). Another method of metastasis in LC involves direct suction, access to the pulmonary vein, and drainage to the left side of the heart (55). Because the JB does not have lymphatic capillaries, hematogenous metastasis is the most prevalent route here. Rich capillary network act as the milieu for the localization of tumour cells. Metastatic foci are more common in red bone marrow than fatty marrow, which allows for greater trapping of metastatic cells due to slow regulation of blood flow control. Red bone marrow also contains growth factors that may help some metastatic tumours cells to colonise faster. JBM is more common in the mandible (posterior area notably the body (premolar-molar region), angle, and ascending ramus) than in the maxilla, owing to the existence of abundant red marrow in the mandible, whereas the maxilla contains mostly fatty marrow (49). Similar results were documented in the current research, with mandibular predominance of JBM and the posterior side was more affected in both jaws.

LC spread to the paranasal sinuses (PNS) is rare (56). Joel *et al*. discovered that the MS were the most common site of metastasis among the PNS (57). The route of metastasis at this region is also suggested to be through hematogenous spread and Batson’s plexus system (58).

TMJ is a rare location of metastasis that usually arises in the late stages of a cancer that is connected with skeletal metastasis. According to Irani *et al*., TMJ and condylar involvement were the least common among the JBM (49). Only 8 cases of metastatic LC affecting the TMJ and condyle were found in the current research. The exact cause of the uncommon occurrence of metastasis in the condylar region is unknown, however is thought to be owing to poor red marrow and a deficit blood flow from the maxillary and temporal arteries. Furthermore, the presence of a bone plate in the condylar region may limit tumour cell proliferation, resulting in decreased tumour cell entrapment (39).

JBM is more difficult to diagnose than soft tissue lesions for the following reasons.

1. They seem similar to squamous cell carcinoma, the most frequent malignant tumour of the jaws.

2. The lesions are placed in the centre of the bone.

3. Unless the disease is advanced, the patient has little subjective symptoms.

4. Lesion radiographs are frequently non-specific.

However, it is possible that the seeming rarity is due in part to a failure to recognise metastatic tumours in the jaws. Furthermore, because the jaws are not frequently inspected at autopsy, some abnormalities may be missed. As a result, the true incidence of metastatic tumours in the jaws may be higher.

Patients with JBM present with asymptomatic lesions to a wide variety of symptoms. The most common symptoms are numb chin syndrome (NCS) or mental nerve neuropathy. Pain, swelling, and tooth loosening are other typical symptoms. Current review revealed that patients presented with variable symptoms (Table  Table 4,  Table 4 cont.-1.). The post-extraction site is regarded as one of the particular JBM sites. Kaugers *et al*., observed a substantial link between trauma and oral metastasis. It backed up the seeding hypothesis, which states that cells from the lungs collect in traumatic sites via sputum, and that these traumatised areas operate as a breeding ground for cancer cells, leading to distant metastases. In our research, we could find only 11 individuals with history of teeth extraction.

JBM is difficult to diagnose since the lesions mimic various inflammatory disorders of the jaw, periapical lesions, odontogenic lesions, malignant jaw tumours. TMJ metastasis can be misinterpreted as TMJ problems. Many cases in the current study were given a preliminary diagnosis of odontogenic tumours, osteomyelitis, malignant tumours of the jaw, salivary gland tumours, and so on. Clinicians must be aware of problems that could result in a misdiagnosis. History of LC could help in the detection of secondary metastatic cancer. JBM via LC is a late indication. 18% of the patients in this research were aware of previous LC, whereas 71% had no such history.

JB metastatic tumours are of high clinical importance because, they may be the only symptom of an undiagnosed underlying malignancy or the first sign of the metastasis. In our study, appx 71% patients had evidence of metastasis as the initial symptom of the disease.

Radiographic characteristics of JBM are not pathognomonic. The type of contact between tumour cells and the bone microenvironment can lead to osteolytic or osteoblastic lesions. Most malignancies are characterised by osteolysis. Osteoblastic lesions are uncommon like caused by prostate cancer (58). Certain tumours can cause reactive new bone development, resulting in a mixed radiopaque and radiolucent lesion. To identify the amount of soft tissue involvement and other sites of distant metastasis in the body, computerized tomography (CT) and magnetic resonance imaging (MRI) are required. 45.4% lesions in the current study manifested as osteolytic, with ill-defined radiolucency.

Histopathological examination is required to provide a conclusive diagnosis of the type of JBM. However, it might be difficult to make an exact diagnosis because these lesions have a varied histological appearance rather than a distinct picture. When the major focus of the primary metastatic site is known, diagnosing the secondary metastasis can be simple. Other tools, such as special staining, immunohistochemistry, and electron microscopy, may be necessary in some circumstances to determine the initial tumor’s nature.

Many new entities of LC have recently been introduced to the World Health Organization (WHO) classification system 2015 (59). Adenocarcinoma has been discovered to be the most prevalent type of LC that metastasizes to the JB. And same was the finding in this study as well. Mucoepidermoid carcinoma is a salivary gland cancer that seldom spreads to the lungs. Only 1 such case has been documented in the current research (60).

Although LC entails multiorgan distant metastases, JB might occasionally be the only site of metastasis. Out of 66 instances in this study, 32 had JB as the only location of LC metastasis, whereas 25 had metastasis to other parts of the body as well such as brain, kidney, adrenal, liver, vertebrae, spine, pelvis, skin, ocular, and skeletal muscles.

JBM treatment and prognosis are determined by the site of genesis and the extent of the disease. Treatment options include biopsy, local excision, chemotherapy, radiotherapy brachytherapy, and/or combination therapy. Commonly used therapeutic aids in this study were radiotherapy, chemotherapy, surgery and combined therapy. Unfortunately, JBM by LC has a bad prognosis with a maximum survival rate of 5 years. Even after treatment, 44% people died, according to the current study. 5 patients had a good prognosis with no signs of recurrence.

Limitations of the current study

One of the limitations of current research was small sample size. Most of studies included were case reports and case series. Population based analysis was not included. We excluded epidemiological, case control studies because we also aimed to evaluate individual features of these metastatic lesions. And in those studies, individual data of patients was not available.

## Conclusions

During the last 60 years (December 1961-December 2021), we found only 66 published cases of LC metastasis to JB, according to our research. These findings suggest that LC metastasis to JB is a rare occurrence. The prognosis was poor with a survival rate of 1 week to 1.5 years. Metastasis of LC predominantly involved mandible than maxilla. The most prevalent type of LC diagnosed was Adenocarcinoma. Because of their resemblance to other jaw problems and late clinical signs, these lesions go unnoticed the majority of the time. Diagnosis of JBM is a challenging task for the clinicians, and pathologists. A thorough examination of the metastatic lesions is required, including a review of the patient’s medical history, clinical presentation, and early diagnosis in order to identify the primary site of metastasis and choose the best course of treatment. More cases need to be published in order to raise awareness of these lesions and gain a better understanding of their characteristics.
